# Origins and consequences of technology acquirement by independent-living seniors: towards an integrative model

**DOI:** 10.1186/s12877-017-0582-5

**Published:** 2017-08-22

**Authors:** S. T. M. Peek, K. G. Luijkx, H. J. M. Vrijhoef, M. E. Nieboer, S. Aarts, C. S. van der Voort, M. D. Rijnaard, E. J. M. Wouters

**Affiliations:** 1 School of Social and Behavioral Sciences, Department of Tranzo, Tilburg University, Tilburg, The Netherlands; 2 Institute of Allied Health Professions, Chair of Health Innovations and Technology, Fontys University of Applied Sciences, Eindhoven, The Netherlands

**Keywords:** Technology adoption, Technology acceptance, Older adults, Seniors, Aging in place, Independent living, Longitudinal research, Qualitative research, Model, Assistive technology

## Abstract

**Background:**

Living independently can be challenging for seniors**.** Technologies are expected to help older adults age in place, yet little empirical research is available on how seniors develop a need for technologies, how they acquire these technologies, and how these subsequently affect their lives. Aging is complex, dynamic and personal. But how does this translate to seniors’ adoption and acceptance of technology? To better understand origins and consequences of technology acquirement by independent-living seniors, an explorative longitudinal qualitative field study was set up.

**Methods:**

Home visits were made to 33 community-dwelling seniors living in the Netherlands, on three occasions (2012–2014). Semi-structured interviews were conducted on the timeline of acquirements, and people and factors involved in acquirements. Additionally, participants were interviewed on experiences in using technologies since acquirement. Thematic analysis was employed to analyze interview transcripts, using a realist approach to better understand the contexts, mechanisms and outcomes of technology acquirements.

**Results:**

Findings were accumulated in a new conceptual model: The Cycle of Technology Acquirement by Independent-Living Seniors (C-TAILS), which provides an integrative perspective on why and how technologies are acquired, and why these may or may not prove to be appropriate and effective, considering an independent-living senior’s needs and circumstances at a given point in time. We found that externally driven and purely desire-driven acquirements led to a higher risk of suboptimal use and low levels of need satisfaction.

**Conclusions:**

Technology acquirement by independent-living seniors may be best characterized as a heterogeneous process with many different origins, pathways and consequences. Furthermore, technologies that are acquired in ways that are not congruent with seniors’ personal needs and circumstances run a higher risk of proving to be ineffective or inappropriate. Yet, these needs and circumstances are subject to change, and the C-TAILS model can be employed to better understand contexts and mechanisms that come into play.

## Background

*“In the end, my mother decided to buy herself an iPad... For years, my suggestion that my mother should get a tablet has fallen on deaf ears. Then, her trusty old PC broke, a friend sang the praises of her own tablet, and the next thing I know, she is Facetiming me.”* [1]

Older adults are often considered ‘laggards’ and ‘resistant’ when it comes to acquiring technology [2, 3]. Yet, the above fragment from a BBC news article titled *“The generation that tech forgot”* [1] demonstrates that certain events and developments in the life of an older adult can trigger the purchase of technologies. The example above also raises several questions. Apparently, two events triggered the purchase of the iPad: the breakdown of the PC, and the recommendation by a friend. If just one of these events had occurred, would the purchase still have taken place? Additionally, why did the older adult not just replace her broken PC, instead of purchasing an iPad? Furthermore, many sons and daughters of older adults are trying to convince their parents to use technologies such as mobile phones, computers and personal alarms [4–6]. What would have happened if the author of the article, and daughter of the older adult, just had given her mother the iPad? Would her mother be just as motivated to use it and take benefit of it?

Understanding the origins and consequences of seniors’ acquirement of technology is important from both a healthcare (demand) and a business (supply) perspective. All around the world the number of older adults is increasing. In the more developed regions, 24% of the population is already aged 60 years or over, and that proportion is projected to reach 29% in 2030 and 33% in 2050 [7]. Globally, the number of people aged 80 years or older is growing even faster. In developed regions, 5% currently is aged 80 years or older. In 2050 this will have doubled to 10% [7]. The inevitable increase of the number of seniors in our society poses challenges as well as opportunities. Looking at healthcare demand, governments are rightfully concerned about the sustainability of current healthcare systems [8]. In response, policy makers aim to enable and facilitate independent living of older adults within the community (i.e., aging in place) [9]. This strategy is expected to avoid expensive institutional care of older adults, and to provide a means to cope with the anticipated shortage of care professionals [8, 10]. As part of this strategy, deploying technology that enables independent living by older adults is considered important [10–14].

From a business perspective, the older consumer market has, in general, long been considered uninteresting and irrelevant [15–18]. However, the trend towards ‘helping older adults to age in place’ has also sparked a wave of new technology products, often developed by start-ups and small and medium-sized enterprises, but also by established multinationals [19–21]. These technologies are referred to as gerontechnology, ambient assisted living technology, smart home technology, or eHealth. They are usually aimed at supporting or enhancing activities of daily living, personal health or safety, mobility, communication, and physical activity [22]. Specific examples include personal alarm systems, vital signs monitoring and fall detection devices, mobile phones specifically designed for seniors, and medication reminders [5, 22]. However, adoption rates of these technologies are reported to be low [22–25]. In general, older adults’ adoption of technology can be described as a *“complex issue that is affected by multiple factors”* [26]. Several studies provide an overview of factors that play a role, including various technology-related beliefs, alternatives to technology use, technology related skills, benefits and costs of technology use, personal characteristics such as health status, and social influences [5, 22, 26, 27]. However, insight in the *interplay and dynamics* between factors is very limited. As noted by others, we still do not know very much about when, how and why community-dwelling older adults acquire technology [19, 28–31]. Additionally, from both a healthcare and business perspective, the ultimate goal is to develop and deploy technologies that contribute to the quality of life of older adults [29, 30, 32]. Since many technologies fail to reach their intended audience, it is important to develop fundamental knowledge on how older adults develop a need for technologies, how they acquire these technologies, and how these technologies subsequently affect their lives.

### Understanding technology acquirements by seniors

Previous research among seniors points to several aspects that need to be taken into account, when aiming to understand the origins and consequences of their technology acquirements. First, the older adult population is highly heterogeneous [33–35]. Within the gerontological literature, there is ample evidence demonstrating increases in physical, sociological and psychological variability with age [36–38]. Therefore, older adults should not be treated and approached as a single homogeneous group [15–17, 29, 33, 39]. Second, as people grow older, they go through changes that affect their need for technologies, as well as their perceptions and responses to technology products [15, 40]. According to consumer psychology literature, older adults do not only vary with regards to their values, attitudes, needs and wants [17], but also with regards to how these are affected by aging, life events, and changes in their social and physical environment [30]. The older people get, the more difficult it becomes to cope with these changes, and the more difficult it becomes to continue to age in place [41]. However, research on the experiences and preferences of independent-living older consumers is scarce. Third, many acquirement decisions of older adults are unlikely to be made in isolation [18, 29]. Previous studies indicate that family members and peers play an important role in older adults’ adoption of technology, particularly by offering advice and support [4–6, 22, 26, 27, 42]. Additionally, relatives may buy technology products for older adults. [4, 27]. However, older adults and relatives do not always see eye-to-eye with regards to the older adults’ need for acquiring technology [4, 5, 27]. Currently, it is unclear how the influence of family and peers during the acquisition process subsequently affects older adults’ use of technologies.

### Research aims

The current study aimed to understand the origins and consequences of technology acquirement by independent-living older adults. We did this by exploring: (1) how and why technologies are acquired by independent-living older adults; and (2) the implications of the ways in which independent-living older adults acquire technologies. In this pursuit, we appreciated that older adults are diverse, that their lives are subject to change, and that their acquirement of technology may be influenced by their family and friends.

## Methods

### Design

To capture both the origins and consequences of technology acquirement, a prospective and explorative longitudinal qualitative field study was carried out [43–45], which involved home visits to independent-living older adults on three occasions (*t*_1_, *t*_2,_ and *t*_3_; 2012–2014). The Ethics Review Board of the Tilburg School of Social and Behavioral Sciences approved the study.

### Sampling

In 2012, a purposive sample of independent-living older adults with different health statuses, living arrangements, and levels of technology experience was recruited. Sources of recruitment were home care providers, a senior volunteer organization, a local tablet computer pilot project, a local shopping center, and word of mouth contacts. Inclusion criteria were: (1) independently living at home, (2) aged 70 or older, (3) Dutch nationality, and (4) no cognitive impairment as measured by the Mini-Mental State Examination (MMSE) [46] using a score of 24 as cutoff [47]. All participants were living in the same medium-sized city in the Netherlands, and one participant was included per household. Potential participants were first handed an information letter, and were telephoned to schedule the first home visit after they expressed an interest in participating in the study. Of the 72 approached individuals, 53 agreed to participate (*N* = 53, *t*_1_). Over the course of the study subsequently 18 and 2 participants dropped out (*N* = 35, *t*_2_; *N* = 33, *t*_3_)_._ Reasons for drop out were: not interested in continuing (*n* = 5), deceased (*n* = 4), somatic health problems (*n* = 4), cognitive impairment (*n* = 2), too busy providing informal care for their partner (*n* = 2), no longer living independently (*n* = 2), and lost contact (*n* = 1). For the study reported here, only individuals who participated in *t*_1_, *t*_2_ and *t*_3_ were included (*N* = 33).

### Data collection

At the beginning of the first visit (*t*_1_), informed consent was acquired. Prior to the second and third visit (*t*_2,_ and *t*_3_), participants were informed by letter on the research project’s progress, and participants were called to schedule a home visit at their convenience. Home visits were performed by pairs of researchers (SP, MN, SA, CvdV, and MR).

The aim of the data collection at *t*_1_ (September – December 2012) was to understand participants’ lives, and their perceptions and attitudes towards technologies.

For this purpose, we performed three types of data collection: (1) background information on educational level, civil status, living arrangement, level of formal and informal care, chronic conditions, subjective health status, frailty as measured by the Tilburg Frailty Indicator (TFI) [48], and cognitive functioning as measured by the Mini-Mental State Examination (MMSE) [46]. Additionally, participants were asked whether they had experienced life events that were meaningful to them in the last 12 months; (2) participants and visiting researchers jointly made a tour through the home, in which an inventory was drawn up of electronic devices. Devices were included if they required electric power in order to function, were intended to be used in or around the home, and could support activities of daily living, personal health or safety, mobility, communication, and physical activity. Ownership and type and frequency of use were recorded; (3) semi-structured interviews were conducted in which participants were interviewed on their perceptions and attitudes toward the devices that were in their home, as well as reasons for ownership and level of use. In particular, we were interested in technologies that were integrated in the daily lives of participants, as well as technologies that were not, or to a lesser extent. Initially, a topic list based on our systematic review of factors influencing acceptance of technology for aging in place was used [22]. As data collection progressed, the topic list was adjusted. Participants were offered a magazine subscription of their choice at the end of the visit. Subscriptions lasted until the end of the study (also for participants who dropped out).

At *t*_2_ (May – July 2013) and *t*_3_ (March – June 2014) data collection was aimed at understanding why participants acquired new devices since *t*_1_, and at investigating participants’ experiences with new devices after acquirement. First, the same type of background information on participants as in *t*_1_ was gathered. Secondly, participants were asked whether they had acquired electronic devices since the last visit. We recorded the date on which each new device was acquired, and the frequency of use at the time of the visit. Lastly, a semi-structured interview on the acquirement of devices was conducted. We were particularly interested in understanding the timeline of acquirements, and the people and factors involved in acquirements. Additionally, participants were interviewed on their experiences in using devices since acquirement, focusing on their satisfaction with the device, and the implications of using it. When a participant had acquired many devices, we selected a number of devices to discuss, aiming to include various types. During the interviews, we took into account the background information that was gathered on each participant, and relevant themes which had emerged in previous interviews with the participant. The topic list used in the interviews was based on the topic list used at *t*_1_, and evolved as data collection progressed. In this stage of the data collection, we made sure that at least one of the two visiting researchers had visited the participant before. All interviews were audiotaped and transcribed verbatim by a professional transcription service.

### Analysis

Qualitative analysis of transcripts entailed two phases. In the first phase, thematic analysis [49, 50] was employed by a pool of six researchers (SP, KL, MN, SA, CvdV, and MR). The thematic analysis process took place during and between all three waves of the data collection, and was supported by the use of qualitative data analysis software (Atlas.ti versions 6 and 7). In this process, we studied transcripts and attached inductive codes to quotations relevant to the research questions. To increase our understanding of the data, all *t*_1_ transcripts, two-thirds of the *t*_2_ transcripts, and one-third of the *t*_3_ transcripts were first coded independently by two different researchers from the pool of six (in alternating pairs). We aimed to have transcripts analyzed by a researcher who was present at the interview, and a researcher who was not, to fuel discussion. The two researchers discussed their analyses, and produced a single coded version of each transcript. Periodically, these coded transcripts were combined into one Atlas.ti file by SP. This file was used in group sessions in which new codes were discussed, and overarching themes of codes were formed. Soon after the analysis of the *t*_1_ transcripts, few new codes were added, which indicated that data saturation was reached with regards to which factors and themes had influenced ownership and level of use of technology. However, in order to understand the dynamics and interplay between these factors and themes over time, an additional phase of data analysis was necessary.

In phase two of the analysis, the dynamics and interplay between factors and themes were analyzed by SP, KL, HV and EW, using a realist approach [51, 52]. Central to this approach is the idea that a specific context (C) can trigger or enable a number of mechanisms (M), and that combinations of C and M lead to certain outcomes of interest (O). This can be explained by the analogy of gun powder; *“When a spark is introduced to gun powder, the chemical composition of gun powder (mechanism) results in an explosion (outcome). However, there are no explosions if the context is not right—damp conditions, insufficient powder, not adequately compact, no oxygen present, duration of heat applied is too short (context)”* [53]. The realist approach is particularly suitable for gaining understanding on how and why outcomes of interest originate, and in what circumstances [54, 55]. As such, this approach is fitting for our study, in which we sought to understand origins (context, mechanisms) and consequences (outcomes) of technology acquirement by independent-living older adults. In using the realist approach, our work focused on distinguishing contexts, mechanisms and outcomes out of the factors and themes that had emerged during the first of phase our qualitative analysis. For this purpose, SP applied constant comparison [56], systematically comparing technology acquirements by each participant, and between participants. In this iterative process, insights and findings were discussed with KL, HV, and EW on a regular basis.

### Member checking

Member checking took place in two ways. First, in order to promote descriptive and interpretative validity [57], a summary of each interview was sent to participants shortly after *t*_1_, *t*_2_ and *t*_3_. On one occasion, a participant felt she was misinterpreted during an interview. This was discussed with the participant, and taken into account during data analysis. On all other accounts, participants had no remarks with regards to the summaries.

Second, as an additional step, extra home visits were made to participants in June and July 2015. The goal here was to promote theoretical validity [57], and the sole purpose of these extra visits was to share and discuss our interpretations of the interview data across the entire study. With participants, we discussed findings that were particular to them, including acquirement patterns and processes. Additionally, we discussed characteristics that were typical to the participant or his or her situation. Furthermore, we illustrated to them how they –in our eyes- differed from other participants. The discussions helped us in shaping our conceptual model. Out of the 33 participants, 25 participated in this final member check. Reasons for not participating were: personal health problems (*n* = 3), deceased (*n* = 3), and lost contact (*n* = 2). All participants recognized themselves very well in our descriptions of them and their acquirements of technology. Participants would sometimes add specifics that were in line with our analysis. These were recorded, but did not alter our conclusions.

## Results

In the following paragraphs, we first describe the characteristics of the sample. Next, we describe the origins (i.e., context and mechanisms) and consequences (i.e., outcomes) of technology acquirements by participants. In our description of the origins of technology acquirement, we discern between the status quo of participants prior to acquirement, decisive developments within that status quo, and acquirement enabling mechanisms. In the last two paragraphs, we describe the number and types of acquirements by participants, and favorable and unfavorable consequences of technology acquirements. In the discussion section, a new conceptual model that captures the aforementioned is presented.

### Sample

The sample consisted of 33 participants who were aged in their seventies and early eighties (Table 1). There were more females than males in the sample, and the majority lived alone. Most participants had attained some form of secondary education and received homecare, although the latter fluctuated during the study. A vast proportion of the participants considered their health good, very good, or excellent, although this number dropped at *t*_*3*_. The participants’ frailty (TFI) score, which potentially could range between 1 and 15, was lowest at *t*_*2,*_ and highest at *t*_*3*_. The participants’ cognitive functioning (MMSE) score, which potentially could range between 0 and 30, remained stable around 28, indicating normal cognitive functioning among participants.

**Table 1:** Sample characteristics (*N* = 33)

	t_1_	t_2_	t_3_
Age: mean ± SD, in years	76.1 ± 3.9^a^	76.6 ± 4.0	77.5 ± 3.9
Gender			
Female: n (%)	20 (60.6)		
Male: n (%)	13 (39.4)		
Education			
None or primary: n (%)	9 (27.3)		
Secondary: n (%)	20 (60.6)		
Higher: n (%)	4 (12.1)		
Living arrangement			
Alone: n (%)	21 (63.6)	22 (66.7)	22 (66.7)
With a partner: n (%)	12 (36.4)	11 (33.3)	11 (33.3)
Receiving home care: n (%)			
Yes: n (%)	19 (57.6)	22 (66.7)	21 (63.6)
No: n (%)	14 (42.4)	11 (33.3)	12 (36.4)
Subjective health			
Good, very good or excellent: n (%)	23 (69.7)	23 (69.7)	20 (60.6)
Fair or poor: n (%)	10 (30.3)	10 (30.3)	13 (39.4)
TFI score^b^: mean ± SD	4.3 ± 2.7	3.8 ± 2.4	4.6 ± 2.6
MMSE score^c^: mean ± SD	28.1 ± 1.5	28.5 ± 1.5	28.2 ± 1.5

^a^During the home visits, one participant mentioned he was 68 years old, and another participant mentioned he was 69 years old. Both participants were not excluded due to ethical considerations

^b^As suggested by Gobbens et al. [48], a Tilburg Frailty Indicator (TFI) score of 5 was used as the cut-off point for frailty

^c^As suggested by Kempen, Brilman and Ormel [47], a Mini-Mental State Examination (MMSE) score of 24 was used as the cut-off point for cognitive impairment

### Status quo prior to acquirement

Looking at the context from which acquirements originated, analysis showed that six major components captured participants’ ownership and use of technology at *t*_1_ (Table 2). Taken together, we label these components the status quo (i.e., the current state of affairs). As will be explained in more detail later, developments in these components at *t*_*2*_ and *t*_*3*_ could induce technology acquirements among participants.

**Table 2:** Major components of the status quo prior to acquirement

Challenges of independent living	
Use of technological and non-technological means	
Internal technology related schemas and attitudes	
External influence of the social network	
External influence of organizations	
Physical environment	

#### Challenges of independent living

Participants mentioned various experienced and/or expected challenges related to living independently. More specifically, participants in various degrees mentioned how important it was for them to stay active, healthy, connected, mobile, independent and/or safe. The need to stay active could entail a number of activities, varying from being able to do housework, to being active in voluntary work. The need to stay connected included the need for social contact with others, but also the need to *“stay in touch with what is going on in the world”* (P14). For participants, wanting to remain independent not only implied being able to look after oneself, but also experiencing freedom to do what you want to do, and not feeling ‘in debt’ towards others such as family members, for example by asking them for help. Concerning the above-mentioned needs, a considerable amount of variation was noticed among participants. First, some participants displayed urgent concerns with regards to meeting experienced or expected challenges, while others mainly linked challenges to other older adults who were ‘worse off’ than they were. Second, while some participants spoke about various needs, other participants’ discussions of needs were restricted to one or two needs that were central to them, and very much on the foreground (i.e., staying safe, healthy).

#### Use of technological and non-technological means

In order to meet their challenges in the domain of independent living, participants employed non-technological and/or technological means (i.e., technology products). Technological means used by participants included assistive devices (e.g., personal alarm buttons and electric lift chairs), home and personal care appliances (e.g., microwave ovens and electric toothbrushes), home automation devices (e.g., remote controlled power sockets and motorized rolling shutters), home fitness equipment (e.g., treadmills and exercise bikes), ICT devices (e.g., laptops and tablets), telephones (e.g., landline phones and feature phones), and transportation devices (e.g., cars and bicycles). These technological means were used to various extents by participants. Some devices were part of participants’ routines, while other devices were owned but used sub optimally, as was often the case with for example personal alarm buttons and fitness equipment. Additionally, the use of technological means regularly competed with the use of other technological means: “*I find my landline phone convenient… I do not want two… A mobile phone and a landline phone, that is too much for me”* (P3). Moreover, the use of technological means competed with the use of non-technological means, for example hiring a housekeeper instead of using a vacuum cleaner, or asking relatives to look something up online in order to avoid personally using a computer. The number and type of means available were dependent on each participant’s specific context. In some cases, participants expressed to be forced to use a technological mean that they were not satisfied with, because they had no alternative.

#### Internal technology related schemas and attitudes

Analysis showed that through interacting with technological means, participants had formed internal technology related schemas and attitudes. Participants’ technology related schemas contained three sets of beliefs. The first set of beliefs was concerned with the properties of technological means. For example, participants had favorable or unfavorable beliefs concerning the reliability, lifespan, power consumption, and costs of purchase and maintenance of technological means. The second set of beliefs entailed the perceived consequences of using a technological mean. These could involve consequences for the participant as well as consequences for others such as relatives. In many cases, participants perceived both positive and negative consequences of using technological means. For example, for a male participant living alone, using a microwave oven meant that he could remain independent because it allowed him to cook his own meals. At the same time, the fact that he could use a microwave also implicated that his children did not invite him as much for dinner as he would like to. Additionally, many participants did not want to start using assistive technology because they anticipated it would make them appear old or frail (a negative consequence). The third and last set of beliefs was concerned with participants’ self-efficacy in using technological means. Participants frequently made references to their (in)ability to use certain types of devices (e.g., ICT devices), and anticipated using them would make them feel frustrated or stressed.

With regards to technology related attitudes, three types of attitudes could be discerned: the participants’ interest in technological means, the participants’ perceived need for technological means, and the participants’ willingness to invest in technological means. Concerning participants’ interest in technological means, participants often spoke in general terms as if they were a technology-minded person: *“I have always loved everything that is technical”* (P9), or a ‘non-technological' person. Whenever participants did not own or use a certain technology (e.g., smartphones or computers), they often stated that they did not perceive a need for it. In these cases, they regularly referred to alternative means that could meet their needs, or they stated that their needs or preferences were not in line with what that particular technology had to offer. The participants’ willingness to invest in technological means entailed both the willingness to commit to a personal effort so that a device could be used, as well as the willingness to invest financially. Some participants were very willing to invest, while others pointed out that they only had a limited amount of energy and money, or were not motivated to try or learn a device: *“Frankly, I do not feel like putting in the effort”* (P29).

#### External influence of the social network

The social network included the participants’ partners, their children, other relatives, and peers. These members of the social network provided participants with advice, and gave practical, financial and/or emotional support. Sometimes, participants mentioned that it was because of the social network that they owned a technological mean, not because they saw a need themselves *“When I got my first stroke, my children told me: mother you have to get a mobile phone! That’s when my daughter gave me one”* (P6). Members of the social network also influenced participants because they were (co)users of technology. For example, participants saw the ways in which others used modern technology such as tablet computers and electric bicycles. Additionally, participants’ use of communication devices was induced and maintained by relatives, who frequently e-mailed, texted or called participants.

#### External influence of the social organizations

Although less frequently mentioned, participants were also influenced by the actions of organizations; technology suppliers and stores, home care providers, and agencies that can provide financial compensation (i.e., insurance companies, municipalities). For example, participants frequently recollected that a special offer by a store triggered them to buy a device, and pointed out how important technical support was to them. Some participants were concerned whether they would continue to be reimbursed for assistive devices that they had become accustomed to use (e.g., hearing aids). Certain policies of home care organizations also influenced the use of assistive technology, but not other types of technology. For example, some participants received information regarding available assistive devices, and were given the opportunity to try out a number of devices.

#### Physical environment

The last component of the status quo was the physical environment. Participants did not like devices that they considered too intrusive (i.e., disrupted the interior of their homes). Additionally, physical circumstances outside of the home such as inaccessible buildings and bad weather conditions sometimes interfered with using mobility aids and means of transport.^1^

### Decisive developments within the status quo

Participants owned an average of 27 devices at *t*_*1*_ *.* Over the course of the study (at *t*_*2*_ and *t*_*3*_), participants on average acquired a total of 3 devices. A total of 93 devices were acquired, of which 60 acquirements (65%) were discussed in semi-structured interviews with participants. Analysis showed that each time an acquirement had occurred, there were decisive developments that had taken place, which in turn triggered acquirement-enabling mechanisms. A total of 16 distinct developments within various components of the status quo could be identified (Table 3).

**Table 3:** Decisive developments within components of the status quo

Component of the status quo	Decisive developments
Challenges of independent living	The older adult’s needs change, causing an already owned technological mean to be less appropriate, or its use increasingly difficult
	The older adult anticipates a future increase in one or more needs
Use of technological and non-technological means	An already owned technological mean with expired warranty breaks down or wears out
	Maintenance costs of an already owned technological mean increase
External influence of the social network	People in the social network ask or advise the older adult to use a new technological mean
	People in the social network use a technological mean that the older adults does not have, and the older adult sees that they are very satisfied with it
	When visiting people in the social network, the older adult tries out a technological mean which he or she does not own
	People in the social network become dissatisfied with the use of a technological mean by the older adult
	A member of the social network acquires a new technological mean, leaving that member with a redundant device
External influence of organizations	A technology supplier or store makes an attractive offer
	Technology suppliers or stores no longer supply a technological mean, rendering it obsolete
	A home care organization distributes a technological mean to all of its clients
	A health professional advises a behavioral change
	A health professional advises the older adult to start using a technological mean
Physical environment	The older adult renovates the home
	The older adult moves house

In some acquirements, there was just one decisive development that took place. For example, the breaking down of a routinely used technological mean: *“The thing broke, so we had to buy a new one”* (P8). In other cases, multiple decisive developments took place, within multiple components of the status quo. For example, a male participant who had recently become single stated he wanted to have more contact with women (a change in his needs/challenges related to independent living). Additionally, he observed how people in his social network used their smartphones to chat and exchange photos with others*: “When I see others, I see how easy and enjoyable it is to do that*” (P24) (external influence of his social network). These two developments ultimately led him to acquiring a smartphone.

In some cases, participants acquired various devices because of various decisive developments. For example, a female participant’s decision to buy a new car (to meet her need of staying mobile), was induced by increased maintenance costs of her old car, and an attractive offer made by her car dealer. The same participant also bought a new laptop. In this case, her decision was induced by her grandchildren who wanted to be able to use Skype, and an increased need to experience new things in life.

In other cases, multiple acquirements were induced by a single decisive development, or a single combination of decisive developments. For example, a participant renovated his home, which led him and his wife to acquire several kitchen appliances. Other participants experienced a decrease in their health status, which led to the acquirement of multiple assistive devices.

### Acquirement enabling mechanisms

When one or more of the aforementioned decisive developments occurred in the life of a participant, one or more acquirement enabling mechanisms were triggered. These acquirement-enabling mechanisms included motivations to acquire, and resources to acquire (Table 4).

**Table 4:** Motivations and resources to acquire

Type of mechanism	Subtypes	Description
Motivations to acquire	Personally needing a solution	The older adult realizes that there is a personal problem (challenge) that needs a solution
	Personally wanting to acquire	A technological mean becomes attractive to the older adult, because of favorable expectations and/or attractive pricing
	Envisioning oneself as a user	The older adult identifies with the users of a technological mean, in terms of personal characteristics and technology-related skills
Resources to acquire	Internal	The effort and money to acquire a technological mean are put in by the older adult, or by the older adult and his or her partner
	External	The effort and money to acquire a technological mean are put in by relatives and/or organizations
	Mixed	The effort and money to acquire a technological mean are put in by a combination of internal and external sources

Three types of acquirement enabling motivations could be discerned. First, participants could be motivated to acquire because decisive developments led them to realizing they had a personal problem that needed a solution. For example, a participant mentioned he realized he needed a mobility solution: *“At night, there are no buses, and on Sundays either, that means I am stuck here”* (P17). Second, participants could be motivated because decisive developments had triggered them into wanting to acquire a certain technology. This type of motivation was activated when a participant became attracted to a technology product because he or she had positive expectations of using it and/or because of attractive pricing of the product: *“It was marked down, a special offer. I said: ‘This is worthwhile’”*(P33). The third type of motivation entailed participants envisioning themselves as future users of a technological mean. This implicated that the participant saw him or herself as eligible to be a user of a technology, and thus part of its group of users. This also meant that the older adult no longer considered him or herself superior to typical users (‘only old and frail people use that’), or inferior to typical users (‘that is something for people who are younger and smarter than me’).

Looking at the resources needed to acquire devices, two types of resources were necessary: an investment of effort to acquire, and an investment of money to acquire. In many cases, the participants themselves, either with or without their partners, put in effort and money. However, participants had a limited amount of both of these resources at their disposal: *“I have had to learn: save money first, then shop”* (P20). Overall, participants appeared selective when it came to investing effort or energy in activities, including purchasing technology.

In other cases, the resources to acquire were provided by external sources, predominantly relatives or care organizations. This implicated that, in these cases, participants themselves did not have to invest effort or money in acquiring a technological mean. Typically, when resources were provided externally, no motivations to acquire were triggered in the older adult. For example, a participant was provided with an assistive device by a health care organization without ever considering it before: *“I never would have bought it myself”* (P6). In a minority of the cases, participants themselves set the external provision of resources in motion. For example, a participant mentioned to her daughter that she was interested in having a smartphone. Subsequently, her daughter selected and ordered a smartphone for her online, without further consulting the participant.

In other instances, the provision of resources was mixed, meaning that effort and money were put in by internal and external sources combined. In these cases, there was a dialogue and/or cooperation between the participant and external sources, and/or an implicit or explicit division of tasks. For example, a participant and her daughter first discussed how and why the participant used her mobile phone infrequently, and subsequently went out and bought a senior phone together. In this case, the participant invested effort and money, and the participant’s daughter invested effort. Cases where an acquirement was partly reimbursed by for example a municipality are also considered to fall under the category of mixed provision of resources.

### Number and types of acquirements by participants

Over the course of the study, the combination of the status quo, decisive developments within the status quo, and enabling mechanisms influenced various types of acquirements (Table 5).

**Table 5:** Types of acquirements

Acquirement type	Description	Occurrences: n (%)
Substitution	Replacement of a technological mean, by an identical technological mean	37 (39.8)
Upgrade	Replacement of a technological mean, by a more advanced or newer variant	14 (15.1)
Familiar addition	Addition of a technological mean, of a type that is already owned and used	27 (29.0)
Novel addition	Addition of a technological mean, of a that not is not already owned and used	15 (16.1)
Total		93 (100)

Out of the 93 technological means that were acquired by participants, nearly 40% were substitutions, meaning a device was replaced by an identical device. In nearly 29% of the cases, acquirements entailed the addition of a technological mean of a familiar type (e.g., an additional kitchen appliance). The addition of a novel, unfamiliar type of technological mean (e.g., first time acquirement of an ICT-device), was less frequent (16%). This also applied to cases in which a technological mean was replaced by a more advanced or newer variant (e.g., replacement of a bicycle by an electric bicycle). These types of acquirements were labeled upgrades, and made up 15% of the acquirements.

The prevalence of these types of acquirements differed between participants (Table 6).

**Table 6:** Prevalence of acquirements per participant: number and types of acquirement

	Number of participants (%)	Number of acquirements	Type(s) of acquirement
Participants who did not acquire any technological means during the study	4 (12.1)	0	N/A
Participants who acquired technological means between *t*_1_ and *t*_2_, *and* between *t*_2_ and *t*_3_	3 (9.1)	1	Substitutions
	7 (21.2)	1 or 2	Upgrades and/or familiar additions
	7 (21.2)	3 to 5	Mix of 3 to 4 types
	2 (6.1)	7 to 9	Substitutions and familiar additions
Participants who acquired technological means between *t*_1_ and *t*_2_, *or* between *t*_2_ and *t*_3_	5 (15.1)	2	Mix of 2 to 3 types
	5 (15.1)	4 to 6	Mix of 2 to 3 types
Total	33 (100)	-	-

Four out of the 33 participants did not acquire any technological means during the study. Of the participants that did acquire technological means, three of them only acquired one device, all substitutions. Seven participants acquired one or two devices (upgrades and/or familiar additions), and seven other participants acquired three to five devices (a mix of three or four types of acquirement). Furthermore, two participants acquired seven to nine devices over the course of the study (either substitutions or familiar additions).

In addition, there were ten participants who only acquired technological means in a single time period, either between the first and second home visit (*t*_1_ – *t*_2_), or between the second and third home visit (*t*_2_ – *t*_3_). Five of these participants acquired two devices, and five acquired four to six devices.

#### Moderating factors affecting number and types of acquirements by participants

Comparison between participants’ acquirements over the course of the study led to the discovery of moderating factors, which influenced the number and types of acquirements by participants.

First, there were personal dispositions that came into play. Some participants were more impulsive than others. This was reflected in the time it took them to make purchase decisions. Furthermore, participants buying few technologies referred to themselves as being economical*: “That is what we are used to: how much does it cost? Isn’t there a cheaper way? That is in our system, being economical”* (P32). Additionally, some participants were more willing to try out new things than others. For example, a participant who just bought herself an iPhone: “*An open-minded person. I want to participate in society. I do not have to be at the forefront... but I want to experience it”* (P14). Lastly, participants differed with regards to how willing they were to ask people in their social network for help in buying technological means. This could lead to the postponement of purchases.

Second, there were situational conditions that influenced the number and types of acquirements by participants. Looking at the role of technology suppliers and stores, participants were more likely to purchase technological means themselves when there was a familiar store nearby that they could go to themselves. Offering home delivery was also mentioned as being important by participants. In some cases, participants found themselves in a buying spree: “*One thing led to another. Beforehand I was not thinking ‘let’s spend some money’”* (P14). This occurred for example when participants were renovating their home, and entered a period of spending. In the case of a buying spree, participants typically mentioned that there was a salesperson who understood their preferences and needs.

There were also conditions which limited or hindered the acquirement of technological means. For example, some participants mentioned they were swamped with choices, once they had decided that they wanted a certain type of device. In these cases, they did not know which model or brand to buy. When this occurred, several participants reverted to buying the same model as people in their social network. Too many options to choose from was also an important reason why participants did not buy devices online. In addition, the social network could limit or delay acquirements. For example, some participants disagreed with their partner on buying devices. Additionally, a participant reported that her children’s assistance had its limits: *“Well, we went to one store. My son told me ‘Mother, you should know that I do not have the time to visit all the stores with you’”* (P6). Furthermore, the participants’ health status could limit the amount of energy they had to engage in acquirements, and it could make other situational conditions more critical (e.g., having a store nearby, availability of help by the social network).

### Favorable and unfavorable consequences of acquirements

After participants acquired technological means, they had various experiences while using them, and new technological means could have various implications for their lives (i.e., their particular status quo’s). For example, some participants were satisfied with their new device and used it routinely to satisfy their needs, while others hardly used a new device and did not express being happy with it.

Analysis showed that favorable and unfavorable consequences of an acquirement (i.e., experiences with the new device and implications for the status quo) were strongly linked to how that acquirement had originated (i.e., the combination of the status quo, decisive developments within the status quo, and enabling mechanisms). This can be illustrated by scenarios that involve both the origins and consequences of acquirements. A total of 36 distinct scenarios could be derived from the interviews. Scenarios included the specific status quo prior to acquirement, decisive developments within that status quo, triggered motivations and resources to acquire, the type of acquirement that occurred, experiences in using the newly acquired technological mean, and implications for the status quo. Moderating factors (i.e., personal dispositions and situational conditions) were not included in these scenarios.

Table 7 shows four typical scenarios of technology acquirement with favorable consequences, and Table 8 shows four typical scenarios of technology acquirements with unfavorable consequences.^2^ Each of these tables contains a scenario in which a device is substituted, a scenario in which a device is upgraded, the addition of a familiar type of device, and the addition of a novel type of device.

**Table 7:** Scenarios of technology acquirement with favorable consequences

#	Origins of acquirement				Acquirement	Consequences of acquirement	
	Status quo prior to acquirement	Decisive developments	Motivations to acquire	Resources to acquire	Type of acquirement	Short-term use and experiences	Implications for the status quo
1	A nutrition challenge is met by a routinely used technological mean (a coffee machine), and alternative means are not considered or used	The older adult moves house, and the technological mean does not fit in the interior of the new home	Personally needing a solution	Internal	Substitution of a coffee machine	Satisfaction and routine use	The status quo prior to acquirement is restored
2	A mobility challenge is met by a routinely used technological mean (a bike). People in the social network use a more advanced variant of the technological mean (e-bikes)	The technological mean breaks down, and its warranty is expired. People in the social network ask or advise the older adult to use a more advanced variant. A store makes an attractive offer	Personally needing a solution, personally wanting to acquire, envisioning oneself as a user	Internal	Upgrade of a bicycle to an electric bicycle	Satisfaction and routine use	The older adult has a more advanced technological mean at his or her disposal, and is using in to meet a challenge
3	One or more assistive devices are routinely used and are meeting a variety of health challenges	Health deteriorates rapidly. The older adult anticipates more health problems in the future	Personally needing a solution, Personally wanting to acquire	Internal	Addition of a familiar type of device; a mobility scooter	Satisfaction and routine use	The older adult has an additional technological mean to meet challenges
4	A health challenge (being overweight) is not met by a technological or non-technological mean	During a checkup, a health professional advises the older adult to start using a technological mean	Personally needing a solution	Mixed	Addition of a novel type of device; fitness equipment	Satisfaction and routine use	A previously unmet challenge is now met by a technological mean. Older adult has a positive experience with a new type of device

**Table 8:** Scenarios of technology acquirement with unfavorable consequences

#	Origins of acquirement				Acquirement	Consequences of acquirement	
	Status quo prior to acquirement	Decisive developments	Motivations to acquire	Resources to acquire	Type of acquirement	Short-term use and experiences	Implications for the status quo
1	A challenge (the need for social contact) is met by a routinely used technological mean (a feature phone^a^), and alternative means are not considered or used	Cognitive decline makes using the technological mean increasingly difficult. The social network becomes dissatisfied with the use of the mean and asks or advises the older adult to replace it	Personally needing a solution	Mixed	Substitution of a feature phone^a^	Low use and satisfaction	The acquirement cannot mitigate the effect of cognitive decline on the status quo
2	A challenge (the need for social contact) is met by a routinely used technological mean (a feature phone^a^). People in the social network use a more advanced variant of the technological mean	The technological mean breaks down, and its warranty is expired	-	External	Upgrade of a feature phone^a^ to a smartphone	Older adult cannot make phone calls, and advanced features are not used. Older adult needs help	Deterioration. Older adult has trouble using the technological mean, which is also not used to its full potential
3	A safety challenge is not met by a technological or non-technological mean	A home care organization distributes personal alarms to all of its clients. People in the social network ask or advise the older adult to use this technological mean	-	External	Addition of a novel type of device; assistive technology	Not satisfied with device. Older adult uses it only at night	Slight improvement. The new technological mean is not used to its full potential
4	One or more kitchen appliances are routinely used, and are meeting challenges related to independent living	A store makes an attractive offer	Personally wanting to acquire	Internal	Addition of a familiar type of device; a kitchen appliance	Not satisfied with device. Use decreases rapidly, then stops	No improvement. Older adult also has had a negative experience with acquiring a new device

^a^A mobile phone that lacks the features of a smartphone such as the ability to download and install apps

As can be seen in Table 7, a substitution (row #1) typically originated from a status quo in which a participant was routinely using a technological mean to satisfy his or her needs, without considering alternative means. In favorable scenarios such as the one displayed in Table 7, substitutions led to the restoration of the status quo prior to acquirement, meaning all was well (i.e., the same) again.

In the event of a typical upgrade (row #2), a participant originally used a technological mean routinely, but at the same time was surrounded by people who used a more advanced variant of that mean. In favorable upgrade scenarios, participants ended up with using a more advanced variant of a technological mean that met their needs. In a number of cases, this also resulted in participants gradually or suddenly ceasing to use the previous (older generation) technological mean.

Looking at the addition of a familiar type of device (row #3), this typically originated from a status quo in which one or more technological means of a similar type were already used to meet challenges. When an older adult added a familiar device, he or she had an additional technological mean at his or her disposal that could help meet challenges.

In contrast to a familiar addition, a novel addition (#row 4) typically originated from situations in which a challenge was not met by a technological (or non-technological) mean. This mostly occurred when health or safety challenges were not met. In favorable novel addition scenarios, acquirement led to the fulfilling of previously unmet needs. As an added benefit, participants had a positive experience with a new type of device. As such, their internal technology schemas were more profoundly affected, in comparison to the other less novel types of acquirements. For example, a female participant who acquired her first ICT-device, a tablet computer: *“I am amazed, you know that? That I have learned how to operate it so quickly, and that I have grown accustomed to it. That I am doing it. I would like to see other nearly 79 year olds do this! Who would have thought?!”* (P30).

While 45 out of the 60 acquirements (75%) were successful in the sense that there were favorable consequences as mentioned above, there were also 15 acquirements (25%) that had unfavorable consequences. As can be seen in four typical unfavorable scenarios in Table 8, acquirement could for example lead to no improvement of the status quo, low satisfaction with the new device, and suboptimal use of the new device. In one scenario (Table 8, row #1), the newly acquired technological mean was simply not ‘powerful’ enough to mitigate the effect of a decisive development (cognitive decline).

In all other scenarios, analysis showed that unfavorable consequences of acquirements were predominantly related to the mechanisms that came into play (i.e., which motivations were triggered and how resources to acquire were provided). Two types of situations increased the chances of unfavorable consequences: (1) ‘externally driven acquirements’ with external or mixed provision of resources, and no or limited triggered motivations to acquire on the part of the older adult, and (2) ‘purely desire-driven acquirements’ with internal provision of resources, and personally wanting to acquire as the only motivation to acquire.

Examples of externally driven acquirements are provided in Table 8. In the first example (row #2), the social network provided a female participant with a smartphone, after her feature phone had broken down. However, the participant’s needs and preferences did not seem to be taken into account in this process. As a result, the participant ended up with a phone she could not use. In the second example (row #3), a home care organization distributed personal alarms to all of their clients, without considering each client’s personal circumstances. This resulted in the suboptimal use of this technological mean by three participants who were all clients of the same home care organization. There was one other participant, who was also a client, who used the personal alarm as intended by the home care organization. This participant was already used to wearing a personal alarm button (i.e., the acquirement was a substitution), in contrast to the other participants.

Looking at desire-driven acquirements (e.g., row #4); these were acquirements in which participants themselves bought a device, solely because their personal want to acquire was triggered, usually by an attractive offer made by a store. In these cases, acquirements were not the result of an unfavorable status quo, or problems that arose as decisive developments. Participants bought a device because they wanted to, not because they really needed to. In these cases, participants felt ‘fooled into it’, and could feel guilty, such as a female participant who bought a laptop: “*Yes, yes, I feel really guilty, for not having used the thing*” (P15). Some participants reported that they would think twice, the next time they would feel tempted to buy something.

## Discussion

**Fig. 1:**
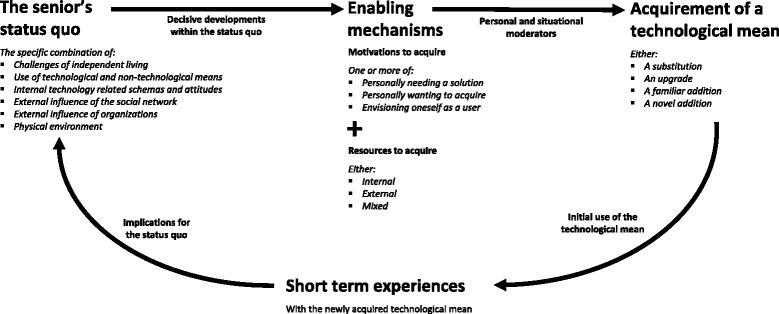
Cycle of Technology Acquirement by Independent-Living Seniors (the C-TAILS model)

The current study sought to provide insight in the origins (i.e., contexts and mechanisms) and consequences (i.e., outcomes) of technology acquirement by independent-living seniors, by applying a realist approach [51, 52]. Our findings can be summarized in a new conceptual model that is presented in Fig. 1: The Cycle of Technology Acquirement by Independent-Living Seniors (C-TAILS). This model is both longitudinal and cyclic. It depicts how various types of technology acquirement originate from an independent-living senior’s specific status quo and decisive developments within that status quo. Subsequently, the model shows how these decisive developments can trigger a number of acquirement enabling mechanisms, and how acquirement can be influenced by personal and situational moderating factors. Lastly, the model depicts the consequences (or implications) of technology acquirement, which are mediated by the seniors’ experiences with the newly acquired technology. As such, the C-TAILS model depicts and integrates both the origins and consequences of technology acquirement by independent-living seniors. It provides an integrated perspective on why and how technological means are acquired, and why these may or may not prove to be appropriate and effective, considering an independent-living senior’s needs and circumstances at a given point in time.

Because of our focus on understanding seniors’ technology acquirement in a natural setting, our study is inherently interdisciplinary [58]. Consequently, our findings and model touch upon and potentially impact several streams of research, including gerontological research, consumer research on buying behavior, and research on acceptance and adoption of technology. Looking more closely at our results, several observations can be made.

First, our results indicate that independent-living seniors’ lives should not be considered static. Rather, independent-living seniors’ lives can be characterized as a changeable system of related components (i.e., the status quo). An important characteristic of the status quo is the balance between seniors’ experienced and/or expected challenges related to independent living, and the technological and non-technological means that they have at their disposal to meet these challenges. As such, our findings are in line with gerontological research on seniors’ perspectives on how to age healthy at home [59], and the continuity theory of normal aging, which poses that seniors strive to preserve and maintain what they have [60]. Our findings are also in agreement with one of the leading models of successful aging, the Selective Optimization with Compensation model (SOC-model) [61]. According to the SOC-model, people select life domains (needs) that are important to them, optimize (acquire) means and resources that facilitate success in these domains, and compensate for losses in these domains (for example by using alternative means) in order to adapt to changes and age successfully [61, 62]. As in the SOC model, our participants varied to the extent that they were conscious of their needs, and acquired means and compensated for the loss of means in active or passive ways. All in all, our model and the SOC model both highlight the importance of taking a broad perspective when it comes to understanding the acquirement and use of a technology by an independent-living senior. It is important to understand the senior’s needs, but also how the technological mean relates to the other technological and non-technological means that the senior has at his or her disposal. A difference with the SOC-model is that our model also takes into account the multifaceted influence of the social network and organizations: these entities can influence the senior’s opinion on technological means, they can provide technological means, and they can be seen as non-technological means that compete with technological means.

Second, our findings show that technology acquirements by independent-living seniors are the result of change(s). One or more decisive developments are vital for acquirement to occur, and these developments activate motivations and resources for acquirement. These findings are in contrast with existing technology acceptance models [63–68], that can be traced back to one seminal theory that originated from social and cognitive psychology: the theory of reasoned action [69]. As result, these models employ variance theory to predict an individual’s intention to use a technology [70]. The perceived usefulness and ease of use of a technology are the two most important predictors within these models [63–68]. However, the aforementioned technology acceptance models do not take into account changes or developments over time [22, 71]. Additionally, the dominance of these variance based models has led researchers to mainly focus on capturing the factors that influence technology use (the what), rather than capturing or understanding the processes that lead to technology use (the why and how) [70, 72–74]. The mechanisms (motivations and resources) that we describe in our model of technology acquirement are similar to previous works on technology acceptance by older adults. These works acknowledge the essential role of perceived benefits and costs of technology, perceived need for technology, support by the social network and the degree to which a technology is in line with the older adult’s self-concept [5, 22, 26, 75, 76]. What is different is that our model also describes the developments and context that lead to the triggering of these mechanisms. As such the current study can be seen as a response to generally unheeded calls for longitudinal research to better understand developments in the process of accepting technology [27, 68, 74, 77]. By describing and incorporating influential developments (changes) as well as relevant context, we hope to contribute to the development of alternative theoretical perspectives, a path recently called for by prominent technology acceptance scholars [71, 72, 78]. More specifically, while perceived need and usefulness are frequently mentioned constructs in literature on technology use by older adults, we feel that there exists little understanding in the literature of what these concepts actually entail for older adults. Why do older adults perceive a technology as useful? And how do older adults develop a need for a technology? Our findings indicate that perceived need for a technology and usefulness of a technology are a function of the older adult’s particular status quo and developments that occur within this status quo. As the current study is explorative, more research is needed to confirm these findings, and to further develop our understanding of the personal relevance of technologies to independent-living seniors.

Third, it is worthwhile to compare our findings to classical process models that describe stages of technology adoption [79] and consumer decision making [80]. According to Rogers [79], individuals who adopt innovations such as technologies pass through the stages of (a) becoming aware of an innovation, (b) forming an attitude toward the innovation, (c) engaging in activities that lead to a decision to adopt or reject the innovation, (d) putting the innovation into use, and (e) seeking confirmation of the decision to adopt. While frequently cited, we are not aware of any empirical studies that have researched these adoption stages among older adults. It is important to note that according to Rogers an innovation is “*an idea, practice or object that is perceived as new by an individual*” [79]. In the case of the current study, this mainly applies to the acquirements that we have labeled ‘novel additions’, and to some extent to acquirements that we have labeled ‘upgrades’. Nearly half of our sample did not experience these two types of acquirements. Additionally, looking at our entire sample, these types of acquirements occurred considerably less frequent than ‘substitutions’ and ‘familiar additions’. Previous research suggests that this may be because deciding on buying novel or more advanced types of products can be difficult for all consumers, and for older consumers in particular [81–87]. Over the years, older adults have gained extensive experience in buying and using certain types of technology (e.g., home appliances, means of transportation), while unfamiliar, novel types of technology (e.g., ICT devices, assistive technology) are often more difficult and stressful to buy and use [82, 88]. Additionally, it has been argued that older adults are more prone to use heuristic/intuitive decision making, which can be characterized as experiential, associative and automatic [31, 89]. This type of decision-making requires limited processing resources, as older adults are able to rely on their internal schemas regarding products, and their affect towards products. As such, this type of decision making seems congruent with buying familiar products [31, 89]. In contrast, buying unfamiliar products may require systematic/elaborative decision making, which is more analytical and resource consuming. This type of decision making involves consciously going through the classic stages of consumer decision making: problem recognition, information search, alternative evaluation, purchase decision, and post-purchase evaluation [31, 89]. The abovementioned research may explain why the classical stages of technology adoption and consumer decision-making cannot readily be fitted to our data; the majority of the technology acquirements by participants are not very novel, and they regularly appear not to be deliberative. For example, many participants ‘automatically’ acquired a similar device because the old device broke down. Additionally, the resources to acquire devices could be provided by older adults themselves, by relatives/organizations, or by a combination of both. This is different from the classical stages of technology adoption and consumer decision-making that are mainly focused on self-adoption and self-buying. Our findings show that in some situations seniors act as independent consumers who make their own choices, while in other situations they are in a more passive role and are provided with means by their environment, and in yet other situations they work together with their environment to acquire means. Returning to the difference between more familiar and more novel types of acquirement, the current study shows that these acquirement types originate from different starting points (i.e. status quo’s). Substitutions and familiar additions originate from situations in which older adults use one or more similar devices that are already satisfying their needs. Upgrades mainly occur in situations in which older adults are surrounded by people who use more advanced variants of an already owned and used device. Interestingly, novel additions are the only type of acquirement that originate from unmet needs. This is in line with a suggestion made by Lunsford and Burnett: “*If the product can meet an otherwise unmet need, the elderly consumer may be able to overcome the risk of buying an unknown good*” [84].

Fourth and last, it seems that the motto ‘the customer is always right’ very much applies to older adults. In line with previous research [90], the vast majority of the technology acquirements by participants were successful, in the sense that they used them, were satisfied with them and they fulfilled their needs. In the literature and by the general public, older adults are often viewed as ‘critical consumers’ [16, 29]. Based on our findings, one could argue that older adults are rightfully critical; their technology acquirements are only unsuccessful when they are ‘externally driven’ or ‘purely desire-driven’. In both situations, our participants felt ‘tricked into’ acquiring a device. Other research suggests that older consumers’ lifetime experience with persuasion attempts may make them relatively resistant to deceptive marketing appeals [91].

### Limitations and suggestions for research

With regards to our data collection and our interactions with participants, several limitations need to be discussed. First, our decision to only interview participants on devices that could support activities of daily living, personal health or safety, mobility, communication, and physical activity may have induced a bias. It is important to note that older adults have more needs than those described in the current study, such as the need to be creative, and the need for personal development. Additionally, older adults also buy nonessential goods such as leisure, entertainment, personal care, and luxury goods [33].

Second, our interview data may have been affected by recall bias since we asked our participants to look back in time in order to construct their acquirements of technology. More specifically, research suggests that older adults’ memory for information tends to skew more positive than that of younger adults [92], causing them to be more satisfied about products than younger consumers across a number of product domains [90]. We have attempted to limit recall bias by only including participants with normal cognitive functioning, by specifically asking participants for positive and negative experiences, and by discussing information put forward by participants that differed from previous interviews with them. The latter occurred rarely, participants seemed to have formed internal storylines of why they acquired technology that remained consistent over the course of the study.

Third, as the study progressed we noticed that participants increasingly considered us trustworthy and opened up more, which facilitated our data collection. On one occasion, a participant disclosed that her acquirement of technology was influenced by an interview. She noted that the interview had caused her to reflect on her technology use, and that this was one of the reasons for her to acquire a number of technologies. According to her, this was due to the topics we addressed, and not a consequence of our style of interviewing. We subsequently asked all other participants whether they felt we were influencing their acquirement and use of technology. All other participants responded that this was not the case.

Looking at our model and findings, there are several areas that could benefit from further research. First, our design focused on exploring why independent-living seniors acquired devices, and not on why they did not. Further research is necessary to understand the context and mechanisms of acquirement processes that are not started, or are aborted. This type of research may also lead to insights on mechanisms that impede acquirement, and mechanisms that limit the enabling mechanisms that we have described.

Second, the current study solely describes older adults’ perceptions of their status quo and developments and mechanisms that led to acquirement. Our model could be expanded by also integrating the perspectives of older adults’ spouses and relatives, as well as care organizations they interact with. It would be interesting to integrate their perspectives on the older adults’ status quo, their views on what mechanisms influence acquirement, and their motivations for providing resources for acquirement. This also would entail collecting more information on the size and nature of older adults’ social networks. As others have pointed out, successful aging in place is socially and collaboratively accomplished [93, 94].

Third, our model could benefit from better specifying the role of affect in technology acquirement processes. While emotions were part of the stories told by participants, we feel that using qualitative methods may not be the best way to capture their precise role. Quantitative research, for example by employing scales developed by Bagozzi [95], may shed light on emotional involvement in the adoption process, by measuring anticipated and anticipatory emotions. Based on our findings, we believe that understanding the role of emotions may be particularly important in novel (unfamiliar) types of acquirement.

Finally, our participants’ views and contexts, as well as their acquirements of technology are likely to be influenced by cultural aspects and the organization of the local and national health care system. Studies in other regions and countries are necessary to determine if our results can be generalized.

### Implications for practice

Independent-living seniors are not only different from each other; they are also different from themselves at different times. This poses problems for those that seek to deploy or implement technologies that aim to support aging in place. It is challenging to present independent-living older adults with relevant and timely offerings.

In dealing with the aforementioned issues, the C-TAILS model can be used to facilitate the deployment and allocation of already existing technological solutions for aging in place. In this pursuit, the C-TAILS model can be used for assessing an older individual’s specific status quo, to understand his or her specific needs and circumstances, in order to determine if technologies in line with these needs would be a welcome addition. Ideally, organizations would over time learn what decisive developments and personal motivations influence their independent-living clients’ technology readiness, and organize the allocation of technological solutions to clients accordingly. Using this strategy, the number of ineffective ‘externally driven’ technology acquirements can be reduced, and older adults can be provided with meaningful and welcome technological means to help them age in place.

Additionally, the C-TAILS model can be of benefit to practice by informing more effective forms of market segmentation, market-research and product design that are more in line with independent-living seniors’ needs and perceptions. Looking at market segmentation, others have noted that dividing a heterogeneous population such as independent-living seniors in subgroups is problematic, even more so if traditional dimensions such as demographics and personal characteristics are used and treated as being static [29, 96–99]. As Dickson noted with regards to segmentation “*A demand results from the interaction of a person with his or her environment, a segmentation perspective that includes both the person and the situation is needed to explain demand*” [99]. In our opinion, and unlike traditional segmentation models, this requires the assignment of more than one segment to each unique older consumer, as the circumstances of that consumer can change. The C-TAILS model can be used to explore and identify these consumer-circumstances segments. This can be done by employing the C-TAILS model in ex ante market research. Ex ante market research frequently employs qualitative methods and aims to shed light on the motivating conditions that ultimately determine the kinds of benefits and attributes that customers will value [100]. Likewise, the C-TAILS model can be used within a contextual design process of technological solutions for independent-living seniors, as the core of this design philosophy is to understand users fundamental intents, desires, and drivers [101].

## Conclusion

Technology acquirement by independent-living seniors may be best characterized as a heterogeneous process with many different origins, pathways and consequences. Furthermore, technologies that are acquired in ways that are not congruent with seniors’ personal needs and circumstances run a higher risk of proving to be ineffective or inappropriate. Yet, these needs and circumstances are subject to change, and the C-TAILS model can be employed to better understand contexts and mechanisms that come into play.

## Abbreviations

C-TAILS: Cycle of Technology Acquirement by Independent-Living Seniors
MMSE: Mini-Mental State Examination
SOC-model: Selective Optimization with Compensation model
TFI: Tilburg Frailty Indicator

## Footnotes

1 For a more elaborative description of the (components of the) status quo, the reader is referred to [27].

2 Due to space constraints, it was decided to report eight prototypical scenarios. The other scenarios can be obtained by contacting the first author.
